# Unscented Kalman Filtering for Single Camera Based Motion and Shape Estimation

**DOI:** 10.3390/s110807437

**Published:** 2011-07-25

**Authors:** Dah-Jing Jwo, Chien-Hao Tseng, Jen-Chu Liu, Hsien-Der Lee

**Affiliations:** 1 Department of Communications, Navigation and Control Engineering, National Taiwan Ocean University, 2 Pei-Ning Rd., Keelung 202-24, Taiwan; 2 National Applied Research Laboratories, National Center for High-Performance Computing, Hsinchu 300, Taiwan; E-Mail: c00how00@nchc.org.tw; 3 Department of Marine Engineering, National Taiwan Ocean University, 2 Pei-Ning Rd., Keelung 202-24, Taiwan; E-Mails: M96660007@mail.ntou.edu.tw (J.-C.L.); hsien@mail.ntou.edu.tw (H.-D.L.)

**Keywords:** motion, shape, optical flow, unscented Kalman filter

## Abstract

Accurate estimation of the motion and shape of a moving object is a challenging task due to great variety of noises present from sources such as electronic components and the influence of the external environment, *etc.* To alleviate the noise, the filtering/estimation approach can be used to reduce it in streaming video to obtain better estimation accuracy in feature points on the moving objects. To deal with the filtering problem in the appropriate nonlinear system, the extended Kalman filter (EKF), which neglects higher-order derivatives in the linearization process, has been very popular. The unscented Kalman filter (UKF), which uses a deterministic sampling approach to capture the mean and covariance estimates with a minimal set of sample points, is able to achieve at least the second order accuracy without Jacobians’ computation involved. In this paper, the UKF is applied to the rigid body motion and shape dynamics to estimate feature points on moving objects. The performance evaluation is carried out through the numerical study. The results show that UKF demonstrates substantial improvement in accuracy estimation for implementing the estimation of motion and planar surface parameters of a single camera.

## 1. Introduction

The problem of estimating positions and velocities of moving features in space leads to the problem of estimating motion and shape parameters of moving features from their corresponding image data observed over time. This is important in various engineering applications, such as robotics and machine vision [1–6]. A dynamical systems approach to machine vision is introduced in [1], in which the problem of motion and shape parameter estimation is described as an inverse problem associated with a pair of coupled Riccati partial differential equations. Identification of the motion and shape parameters using the estimation technique was discussed in [5,6]. A CCD camera with a laser range finder mounted on a mobile robot for better motion and shape parameters identification can be seen in [5]. A sliding mode approach was proposed to estimate the motion of a moving body with the aid of a CCD camera [6]. For performance improvement, various estimation techniques have been adopted to reduce the noise. The algebraic methods yielded feasible results, but they were computationally unrealistic. The Kalman filter method is used in the estimation of the motion parameters to reduce the effect of the measurement errors which are inevitable in the real world. The Kalman filter (KF) method [7] for estimation of the 3D camera motion in imagine sequences for the applications to the video coding system is proposed by Kim *et al.* [8]. Kano *et al.* [9] performed a numerical study and compared an extended Kalman filter (EKF) based recursive algorithm with a non-recursive algebraic method for estimating motion and planar surface parameters.

The KF is theoretically attractive because it has been shown to be the one that minimizes the variance of the estimation mean square error (MSE). The nonlinear filter is used for nonlinear dynamics and/or nonlinear measurement relationships. The problem of estimating the state variables of the nonlinear systems may be solved using the nonlinear version of the Kalman filter. The most popular form is the EKF. The fact that EKF highly depends on a predefined dynamics model forms a major drawback. To achieve good filtering results, the designers are required to have the complete *a priori* knowledge on both the dynamic process and measurement models, in addition to the assumption that both the process and measurement are corrupted by zero-mean Gaussian white sequences. If the input data does not reflect the real model, the estimates may not be reliable.

Similar to the EKF, the unscented Kalman filter (UKF) [10–17] focuses on approximating the prediction probability characteristics and use the standard minimum mean square error estimator. The UKF has been developed in the context of state estimation of dynamic systems as a nonlinear distribution (or densities in the continuous case) approximation method. The UKF is superior to EKF not only in theory but also in many practical situations. The algorithm performs the prediction of the statistics with a set of carefully chosen sample points for capturing mean and covariance of the system. These sample points are sometimes referred to as the sigma points employed to propagate the probability of state distribution. The basic premise behind the UKF is it is easier to approximate a Gaussian distribution than it is to approximate an arbitrary nonlinear function. Instead of linearizing using Jacobian matrices as in the EKF and achieving first-order accuracy, the UKF can capture the states up to at least second order by using a deterministic sampling approach to capture the mean and covariance estimates with a minimal set of sample points. The deterministic sampling based UKF with applications on estimation of rigid body motion and shape dynamics are presented to estimate the feature points on the moving object. Results obtained shows that UKF is able to provide more accurate and reliable estimation accuracy of the object. Investigation of the UKF approach to the motion and shape estimation problem has not been seen in the literature.

This paper is organized as follows. In Section 2, preliminary background on rigid body motion is reviewed. The shape dynamics and optical flow dynamics are discussed in Sections 3 and 4, respectively. The unscented Kalman filter is introduced in Section 5. Results and Discussion are given in Section 6. Conclusions are given in Section 7.

## 2. Rigid Body Motion

**Figure 1.**
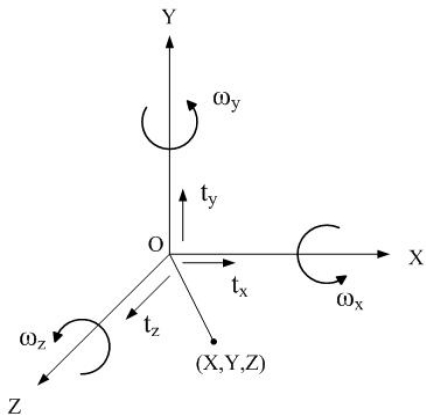
Illustration of the world coordinate system [3].

The mathematical description of a 3D point undergoing a rigid transformation about the camera axes is given as follows. Let *ω_x_*, *ω_y_* and *ω_z_* represent the angle of rotation about the *X*, *Y* and *Z* axes, respectively. Figure 1 illustrates the world coordinate system. An arbitrary rotation **R** can be represented by successive rotations around the *X*, *Y* and *Z* axes, respectively, as:

(1) $$R=\left[\begin{array}{ccc} 1 & 0 & 0\\ 0 & \text{cos}(\omega_{x}) & -\text{sin}(\omega_{x})\\ 0 & \text{sin}(\omega_{x}) & \text{cos}(\omega_{x}) \end{array}\right]\left[\begin{array}{ccc} \text{cos}(\omega_{y}) & 0 & \text{sin}(\omega_{y})\\ 0 & 1 & 0\\ -\text{sin}(\omega_{y}) & 0 & \text{cos}(\omega_{y}) \end{array}\right]\left[\begin{array}{ccc} \text{cos}(\omega_{z}) & -\text{sin}(\omega_{z}) & 0\\ \text{sin}(\omega_{z}) & \text{cos}(\omega_{z}) & 0\\ 0 & 0 & 1 \end{array}\right]$$

Assuming infinitesimal rotations, the zeroth order terms of the Taylor series expansion of the trigonometric functions and cos provide the following approximations:

(2) cos(θ)≈1,         sin(θ)≈θ

Using the approximations in Equation (2), **R** can be approximated in the skew-symmetric matrix form in terms of angular velocity:

(3) $$R\approx \left[\begin{array}{ccc} 1 & 0 & 0\\ 0 & 1 & -\omega_{x}\\ 0 & \omega_{x} & 1 \end{array}\right]\left[\begin{array}{ccc} 1 & 0 & \omega_{y}\\ 0 & 1 & 0\\ -\omega_{y} & 0 & 1 \end{array}\right]\left[\begin{array}{ccc} 1 & -\omega_{z} & 0\\ \omega_{z} & 1 & 0\\ 0 & 0 & 1 \end{array}\right]\approx \left[\begin{array}{ccc} 1 & -\omega_{z} & \omega_{y}\\ \omega_{z} & 1 & -\omega_{x}\\ -\omega_{y} & \omega_{x} & 1 \end{array}\right]$$

Let the vector **T** = [*t_x_* *t_y_* *t_z_*]^T^ represent the translational velocity, where the elemental components *t_x_*, *t_y_*, and *t_z_* represent the translational velocities in the *X*, *Y* and *Z* directions, respectively. The velocity vector **V** = [*Ẋ Ẏ Ż*]^T^ of a point in the world coordinates **P** = [*X Y Z*]^T^ with respect to camera coordinates undergoing rigid transformation is represented as:

(4) $$\left[\begin{array}{c} \dot{X}\\ \dot{Y}\\ \dot{Z} \end{array}\right]=\left[\begin{array}{ccc} 0 & -\omega_{z} & \omega_{y}\\ \omega_{z} & 0 & -\omega_{x}\\ -\omega_{y} & \omega_{x} & 0 \end{array}\right]\left[\begin{array}{c} X\\ Y\\ Z \end{array}\right]+\left[\begin{array}{c} t_{x}\\ t_{y}\\ t_{z} \end{array}\right]$$

which may be written in matrix form:

V=(R−I)P+T

where **I** represents a 3 × 3 identity matrix. By defining [−*ω_z_* *ω_y_* −*ω_x_*]*^T^* as [*ω*_1_ *ω*_2_ *ω*_3_]*^T^* ; and [*t_x_* *t_y_* *t_z_*]*^T^* as [*b*_1_ *b*_2_ *b*_3_]*^T^*, Equation (4) can be represented as:

(5) V=ΩP+b

where **b** = [*b*_1_ *b*_2_ *b*_3_]*^T^* and:

(6) $$\Omega \equiv \left[\begin{array}{ccc} 0 & \omega_{1} & \omega_{2}\\ -\omega_{1} & 0 & \omega_{3}\\ -\omega_{2} & -\omega_{3} & 0 \end{array}\right]$$

Consider the dynamical system Equation (5) where it is assumed that [*ω*_1_ *ω*_2_ *ω*_3_]*^T^* ≠ 0. If:

(7) b∈ImΩ

it is easily seen that Im**Ω** is a plane in ℜ^3^ given as:

(8) $$\text{Im}\Omega =\left\{x\in R|\omega^{T}x=0\right\}$$

where:

(9) $$\omega ={\left[\omega_{3}-\omega_{2}\;\omega_{1}\right]}^{T}$$

## 3. Shape Dynamics

The motion field describing the motion of individual points on the surface might undergo a change in shape. The dynamical system which describes the changing shape of the surface is called the shape dynamics. Let (*X*, *Y*, *Z*) be the world coordinate frame wherein we have a surface defined by:

(10) Z=S(X,Y,t)

Assume that *S* is smooth enough so that its derivatives with respect to each of the variables are defined everywhere. The motion field is assumed to be described by:

(11) $$\dot{X}=f\left(X,Y,Z\right),\;\dot{Y}=g\left(X,Y,Z\right),\;\dot{Z}=h\left(X,Y,Z\right)$$

How the surface as in Equation (10) moves as points on the surface move following the motion field as in Equation (11) can be described by the quasi-linear partial differential equation called the “shape dynamics”:

(12) $$\frac{\partial S}{\partial t}+f\left(X,Y,Z\right)\frac{\partial S}{\partial X}+g\left(X,Y,Z\right)\frac{\partial S}{\partial Y}=h\left(X,Y,Z\right)$$

Consider the initial condition:

(13) S(X,Y,0)=ϕ(X,Y)

The pair of Equations (12) and (13) constitutes an example of a Riccati partial differential equation. The surface defined by Equation (10) is assumed to be a plane described by:

(14) Z=pX+qY+r

where *p*, *q*, *r* are shape parameters that are time varying as a result of the motion field. The vectors (*X*, *Y*, *Z*) and (*p*, *q*, *r*) are written in terms of homogeneous coordinates (*X̄ Ȳ Z̄ W̄*):

(15) $$X=\frac{\bar{X}}{\bar{W}}\;\;\;Y=\frac{\bar{Y}}{\bar{W}}\;\;\;Z=\frac{\bar{Z}}{\bar{W}}$$

and:

(16) $$p=\frac{\bar{p}}{\bar{s}}\;\;\;q=\frac{\bar{q}}{\bar{s}}\;\;\;r=\frac{\bar{r}}{\bar{s}}$$

where *W̄* = (*X*^2^ + *Y*^2^ + *Z*^2^)^1/2^. Equation (14) can then be rewritten as:

(17) $$\bar{p}\bar{X}+\bar{q}\bar{Y}-\bar{s}\bar{Z}+\bar{r}\bar{W}=0$$

Using Equation (15), Equation (5) can be written as:

(18) $$\frac{d}{\mathrm{dt}}\left[\begin{array}{l} \bar{X}\\ \bar{Y}\\ \bar{Z}\\ \bar{W} \end{array}\right]=\left[\begin{array}{cc} \Omega & b\\ 0 & 0 \end{array}\right]\left[\begin{array}{l} \bar{X}\\ \bar{Y}\\ \bar{Z}\\ \bar{W} \end{array}\right]$$

The shape dynamics can be obtained by differentiating Equation (17) with respect to time t:

(19) $$\frac{d}{\mathrm{dt}}\left[\begin{array}{c} \bar{p}\\ \bar{q}\\ -\bar{s}\\ \bar{r} \end{array}\right]=\left[\begin{array}{cc} \Omega & 0\\ -b^{T} & 0 \end{array}\right]\left[\begin{array}{c} \bar{p}\\ \bar{q}\\ -\bar{s}\\ \bar{r} \end{array}\right]$$

where *p*(*t*), *q*(*t*) and *r*(*t*) are the shape parameters to be discussed in Section 4.

## 4. Optical Flow Dynamics

**Figure 2.**
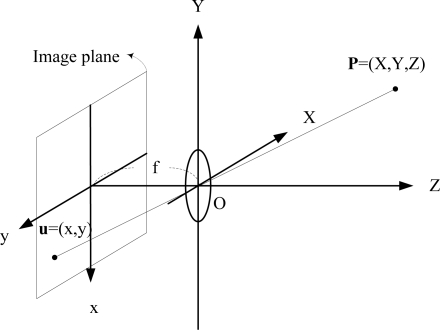
The relationship between an image point **u** = (*x*, *y*) and a scene point **P** = (*X*, *Y*, *Z*).

As shown in Figure 2, the 3D vector **P** = (*X*, *Y*, *Z*) is assumed to be observed via perspective projection onto a plane parallel to the (*x*, *y*) axes and located at *Z* = 1 by defining the relationship between an image point *u* = (*x, y*) and a scene point (*X*, *Y*, *Z*) given by:

(20) $$x=\frac{X}{Z},\;\;y=\frac{Y}{Z}$$

The focal length *f* = 1 is usually used without loss of generality. With the relation given by Equation (5), differentiating Equation (20) leads to the relations:

(21) $$\dot{x}=\frac{Z\dot{X}-X\dot{Z}}{Z^{2}}=\frac{\dot{X}}{Z}-x\frac{\dot{Z}}{Z}=\left(\omega_{1}y+\omega_{2}\right)-x\left(-\omega_{2}x-\omega_{3}y\right)+\frac{b_{1}}{Z}-x\frac{b_{3}}{Z}$$

and:

(22) $$\dot{y}=\frac{Z\dot{Y}-Y\dot{Z}}{Z^{2}}=\frac{\dot{Y}}{Z}-y\frac{\dot{Z}}{Z}=\left(-\omega_{1}x+\omega_{3}\right)-y\left(-\omega_{2}x-\omega_{3}y\right)+\frac{b_{2}}{Z}-y\frac{b_{3}}{Z}$$

From Equations (14) and (20), we have:

(23) $$\frac{1}{Z}=\frac{1-px-qy}{r}$$

and hence the optical flow equations are given by:

(24) $$\dot{x}=\omega_{2}+\omega_{1}y+\omega_{2}x^{2}+\omega_{3}xy+\left(c_{1}-xc_{3}\right)\left(1-px-qy\right)$$

(25) $$\dot{y}=\omega_{3}-\omega_{1}x+\omega_{2}xy+\omega_{3}y^{2}+\left(c_{2}-yc_{3}\right)\left(1-px-qy\right)$$

where *c_i_* = *b_i_*/*r*, *i* = 1,2,3.

Equations (24) and (25) denote the optical flow equations. Even when the motion field is time invariant, the parameters of the optical flow equations could be time-varying due to the fact that the shape parameters are changing in time as a result of motion field described by Equation (11). As *t* → ∞, we have:

(26) $$p(t)\to \frac{\omega_{3}}{\omega_{1}},\;\;q(t)\to -\frac{\omega_{2}}{\omega_{1}},\;\;r(t)\to \frac{\mathrm{bt}}{\omega_{1}},\;\;c_{i}\to \frac{b_{i}\omega_{1}}{\mathrm{bt}},\;\;i=1,2,3$$

In particular, if **b** ∈ ImΩ, the shape parameters *p*(*t*), *q*(*t*) and *r*(*t*) are periodic functions satisfying the Riccati Equation [2,9]:

(27) $$\begin{array}{l} \dot{p}=-\omega_{2}(1+p^{2})+\omega_{1}q-\omega_{3}pq\\ \dot{q}=-\omega_{3}(1+q^{2})-\omega_{1}q-\omega_{2}pq\\ \dot{r}=b_{3}-b_{1}p-b_{2}q-r(\omega_{3}q+\omega_{2}p) \end{array}$$

which is parameterized by a total of six motion parameters and three initial conditions on shape parameters. Therefore, a total of nine parameters need to be adopted for describing the shape dynamics. The Riccati equation propagates in time the relationship between coordinates X, Y, and Z expressed via the surface described by Equation (10).

Once the modeling of shape dynamics and optical flow dynamics is accomplished, the estimation algorithm can be employed for implementing the estimation of motion and planar surface parameters. In addition to the algebraic methods, the recursive estimation algorithms are applicable. The recursive algorithm presented by Kano *et al.* [9] is an EKF-based algorithm. The motivation of the paper is to carry out the UKF-based approach, which has not been seen in the literature for the motion and shape estimation problem. A brief introduction of the UKF is provided in Section 5.

## 5. The Unscented Kalman Filter

The UKF is a nonlinear filter which deals with the case governed by the nonlinear stochastic difference equations:

(28a) $$x_{k+1}=f_{k}(x_{k})+w_{k}$$

(28b) $$z_{k}=h_{k}(x_{k})+v_{k}$$

where the state vector **x***_k_* ∈ ℜ*^n^*, process noise vector **w***_k_* ∈ ℜ*^n^*, measurement vector **z***_k_* ∈ ℜ*^m^*, and measurement noise vector **v***_k_* ∈ ℜ*^m^*. In Equation (28), both the vectors **w***_k_* and **v***_k_* are zero mean Gaussian white sequence having zero crosscorrelation with each other:

(29) $$E\left[w_{k}w_{i}{}^{T}\right]=\{\begin{array}{c} Q_{k},\;\;\;i=k\\ 0,\;\;\;\;i\neq k \end{array};\;\;E[v_{k}v_{i}{}^{T}]=\{\begin{array}{c} R_{k},\;\;\;i=k\\ 0,\;\;\;\;i\neq k \end{array};\;\;E[w_{k}v_{k}{}^{T}]=0\;\;\;\mathrm{for}\;\;\mathrm{all}\;i\;\;\mathrm{and}\;k$$

where **Q***_k_* is the process noise covariance matrix, **R***_k_* is the measurement noise covariance matrix.

### 5.1. The Unscented Transformation

**Figure 3.**
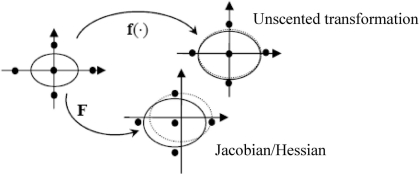
Illustration of properties of UKF and EKF, Reproduced with permission from Yong Li [14].

The first step in the UKF is to sample the prior state distribution, *i.e.*, generate the sigma points through the unscented transformation (UT). Figure 3 illustrates the true means and covariances as compared to those obtained by the mapping of the UKF *versus* that of the EKF. The dot-line ellipse represents the true covariance. The UKF is implemented through the transformation of the nonlinear function **f**(·), shown as the solid-line ellipse on the top portion of the figure; the EKF is accomplished through the Jacobian **F** = ∂**f/**∂**x**, shown as the solid-line ellipse at the bottom portion of the figure. The UKF approach estimates are expected to be closer to the true values than the EKF approach.

Several UT’s are available. One of the popular approaches is the scaled unscented transformation [15–17]. Consider an *n* dimensional random variable **x**, having the mean **x̂** and covariance **P**, and suppose that it propagates through an arbitrary nonlinear function **f**. The unscented transform creates 2*n* +1 sigma vectors **X** (a capital letter) and weighted points, given by:

(30a) $$X^{\left(0\right)}=\hat{x}$$

(30b) $$X^{\left(i\right)}=\hat{x}+{\left(\sqrt{(n+\lambda )P}\right)}_{i}^{T},\;i=1,...,n$$

(30c) $$X^{\left(i+n\right)}=\hat{x}-{\left(\sqrt{(n+\lambda )P}\right)}_{i}^{T},\;i=1,...,n$$

where ${\left(\sqrt{(n+\lambda )P}\right)}_{i}$ is the *i* th row of the matrix square root. $\sqrt{(n+\lambda )P}$ can be obtained from the lower-triangular matrix of the Cholesky factorization; *λ* = *α*^2^(*n* + *κ*) − *n* is a scaling parameter; *α* determines the spread of the sigma points around **x̑** and is usually set to a small positive (e.g., 1*e* − 4 ≤ *α* ≤ 1); κ is a secondly scaling parameter (usually set as 0); *β* is used to incorporate prior knowledge of the distribution of **x̄** (when **x** is normally distributed, *β* = 2 is an optimal value); $W_{i}^{\left(m\right)}$ is the weight for the mean associated with the i*th* point; and $W_{i}^{\left(c\right)}$ is the weigh for the covariance associated with the i*th* point:

(31a) $$W_{0}^{\left(m\right)}=\frac{\lambda }{\left(n+\lambda \right)}$$

(31b) $$W_{0}^{\left(c\right)}=W_{0}^{\left(m\right)}+(1-\alpha^{2}+\beta )$$

(31c) $$W_{i}^{\left(m\right)}=W_{i}^{\left(c\right)}=\frac{1}{2(n+\lambda )},\;\;i=1,...,2n$$

The sigma vectors are propagated through the nonlinear function to yield a set of transformed sigma points:

(32) $$y_{i}=f(X^{\left(i\right)}),\;\;i=0,...,2n$$

The mean and covariance of **y***_i_* are approximated by a weighted average mean and covariance of the transformed sigma points as follows:

(33) $${\bar{y}}_{u}=\sum_{i=0}^{2n}W_{i}^{\left(m\right)}y_{i}$$

(34) $${\bar{P}}_{u}=\sum_{i=0}^{2n}W_{i}^{\left(c\right)}(y_{i}-{\bar{y}}_{u}){\left(y_{i}-{\bar{y}}_{u}\right)}^{T}$$

### 5.2. The Unscented Kalman Filter

**Figure 4.**
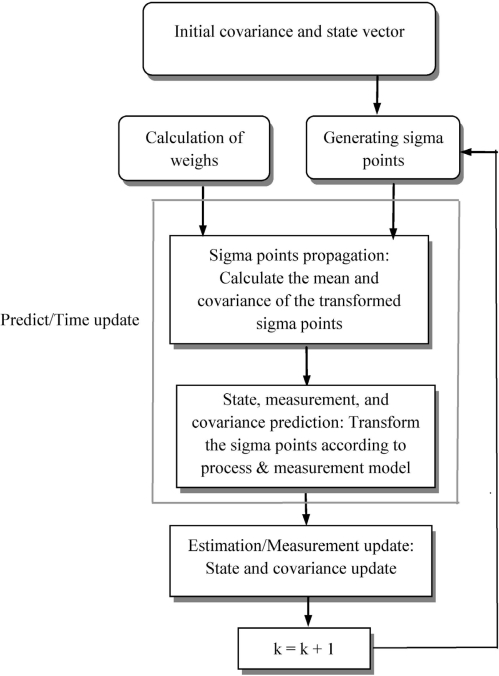
High level of operation of the unscented Kalman filter.

A high level of operation of the unscented Kalman filter is shown in Figure 4. To look at the detailed algorithm of the UKF, firstly, the set of sigma points are created by Equation (30). After the sigma points are generated, the time update (prediction step) of the UKF involves the following steps:

(35) $${\left(\zeta_{k}^{-}\right)}_{i}=f(X_{k-1}^{\left(i\right)}),\;\;i=0,...,2n$$

(36) $${\hat{x}}_{k}^{-}=\sum_{i=0}^{2n}W_{i}^{\left(m\right)}{\left(\zeta_{k}^{-}\right)}_{i}$$

(37) $$P_{k}^{-}=\sum_{i=0}^{2n}W_{i}^{\left(c\right)}[{\left(\zeta_{k}^{-}\right)}_{i}-{\hat{x}}_{k}^{-}]{\left[{\left(\zeta_{k}^{-}\right)}_{i}-{\hat{x}}_{k}^{-}\right]}^{T}+Q_{k-1}$$

(38) $${\left(Z_{k}^{-}\right)}_{i}=h({\left(\zeta_{k}^{-}\right)}_{i})$$

(39) $${\hat{z}}_{k}^{-}=\sum_{i=0}^{2n}W_{i}^{\left(m\right)}{\left(Z_{k}^{-}\right)}_{i}$$

The measurement update (correction) step of the UKF involves the following steps:

(40) $$P_{\mathrm{zz}}=\sum_{i=0}^{2n}W_{i}^{\left(c\right)}[{\left(Z_{k}^{-}\right)}_{i}-{\hat{z}}_{k}^{-}]{\left[{\left(Z_{k}^{-}\right)}_{i}-{\hat{z}}_{k}^{-}\right]}^{T}+R_{k}$$

(41) $$P_{\mathrm{xz}}=\sum_{i=0}^{2n}W_{i}^{\left(c\right)}[{\left(\zeta_{k}^{-}\right)}_{i}-{\hat{x}}_{k}^{-}]{\left[{\left(Z_{k}^{-}\right)}_{i}-{\hat{z}}_{k}^{-}\right]}^{T}$$

(42) $$K_{k}=P_{\mathrm{xz}}P_{\mathrm{zz}}^{-1}$$

(43) $${\hat{x}}_{k}={\hat{x}}_{k}^{-}+K_{k}(z_{k}-{\hat{z}}_{k}^{-})$$

(44) $$P_{k}=P_{k}^{-}-K_{k}P_{\mathrm{zz}}K_{k}^{T}$$

The samples are propagated through true nonlinear equations, which can capture the states up to at least second order.

## 6. Results and Discussion

Simulation experiments have been carried out to evaluate the performance of the UKF approach in comparison with the conventional EKF approach for estimating motion and shape parameters. Computer code was developed using MATLAB. It is assumed that, at each time instant *k*, a set of three feature points (*x_i,k_, y_i,k_*), *i* = 1, 2, 3 are observed. The state vector **x**_k_ is given by:

(45) $$x_{k}={\left[\omega_{1,k}\;\;\omega_{2,k}\;\;\omega_{3,k}\;\;c_{1,k}\;\;c_{2,k}\;\;c_{3,k}\;\;p_{k}\;\;q_{k}\;\;x_{1,k}\;\;y_{1.k}\;\;x_{2,k}\;\;y_{2,k}\;\;x_{3,k}\;\;y_{3,k}\right]}^{T}$$

The associated state equation in discretized form is given by:

(46) $$x_{k+1}=f(x_{k})$$

which is constituted from the set of Equations (24,25) and (27), as follows:

(47) $$\begin{array}{l} p_{k+1}=(\omega_{1,k}q_{k}-\omega_{2,k}-\omega_{2,k}p_{k}^{2}-\omega_{3,k}p_{k}q_{k})\Delta t+p_{k}\\ q_{k+1}=(-\omega_{1,k}p_{k}-\omega_{3,k}-\omega_{2,k}p_{k}q_{k}-\omega_{3}q_{k}^{2})\Delta t+q_{k}\\ r_{k+1}=(b_{3,k}-b_{1,k}p_{k}-b_{2,k}q_{k}-r_{k}(\omega_{3,k}q_{k}+\omega_{2,k}p_{k}))\Delta t+r_{k} \end{array}$$

(48) $$\begin{array}{l} x_{k+1}=(\omega_{2,k}+\omega_{1,k}y_{k}+\omega_{2,k}x_{k}^{2}+\omega_{3,k}x_{k}y_{k}+(c_{1,k}-x_{k}c_{3,k})(1-p_{k}x_{k}-q_{k}y_{k}))\Delta t+x_{k}\\ y_{k+1}=(\omega_{3,k}-\omega_{1,k}x_{k}+\omega_{2,k}x_{k}y_{k}+\omega_{3,k}y_{k}^{2}+(c_{2,k}-y_{k}c_{3,k})(1-p_{k}x_{k}-q_{k}y_{k}))\Delta t+y_{k} \end{array}$$

(49) $$c_{i,k}=b_{i,k}/r_{k}$$

along with the random walk models for the angular velocities *ω*_*i*,*k*+1_ = *ω*_*i*,*k*1_, *i* = 1, 2, 3. Let **z***_k_* ∈ ℜ^6^ represent the observation vector, the observation equation can then be written as:

(50) $$z_{k}={Hx}_{k}+v_{k}$$

where **v***_k_* is assumed to be a zero mean Gaussian white sequences noise with covariance **R** = σ^2^**I**_6_(σ = 0.01). The elements of the measurement model **H**_2_*_n_*_×14_ is defined by:

$$H=[0_{2n\times 8}\;\;I_{2n}],\;\;n=3$$

The process noise covariance matrix is given by **Q** = 10^−10^ × **I**_14_ and the parameters utilized in the UKF are given as follows: *α* = 1*e* − 4, *β* = 2, *κ* = 0. The sigma points capture the same mean and covariance irrespective of the choice of matrix square root which is used. The numerical efficient and stable method such as the Cholesky factorization has been used in obtaining the sigma points.

Experiment was conducted on a simulated three feature points: (2.5,2.5), (1.5,0.5) and (1.0,1.5) locations. The motion and shape parameters Ω is set as (*ω*_1_, *ω*_2_, *ω*_3_) = (−0.2, 0.1, −0.1), with initial values for shape parameters are (*p*_0_, *q*_0_, *r*_0_) = (0.1, 0.1, 2.0). For comparative purposes, the following two cases are considered [2].

$$\begin{array}{l} \text{Case}1:b={\left(\begin{array}{ccc} 0.1 & 0.1 & -0.1 \end{array}\right)}^{T}\in \text{Im}\Omega \\ \text{Case}2:b={\left(\begin{array}{ccc} 0.1 & 0.1 & 0.1 \end{array}\right)}^{T}\notin \text{Im}\Omega \end{array}$$

**Figure 5.**
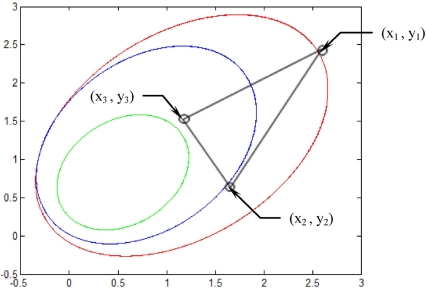
Sample trajectories for the three feature points on image plane for the case **b** ∈ ImΩ.

**Figure 6.**
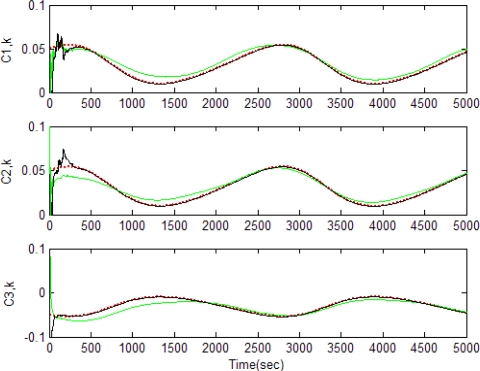
Estimation results for parameters *c*_1_, *c*_2_ and *c*_3_ for the three feature points for the case **b** ∈ ImΩ (true-in red; EKF-in green; UKF-in black).

**Figure 7.**
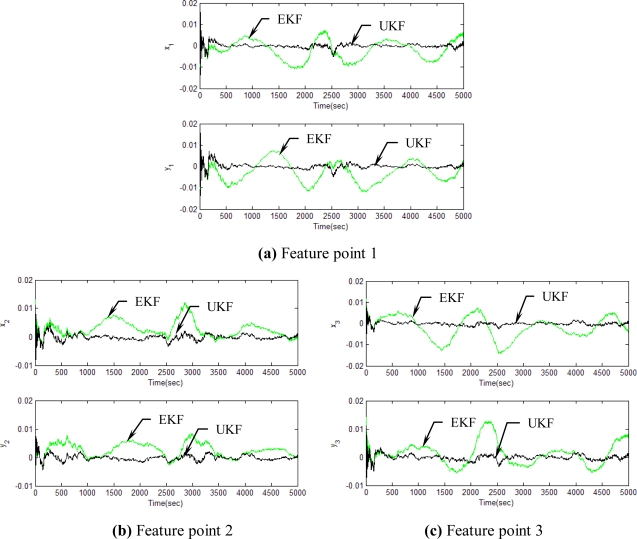
Estimation errors for the three feature points for the case **b** ∈ ImΩ.

**Figure 8.**
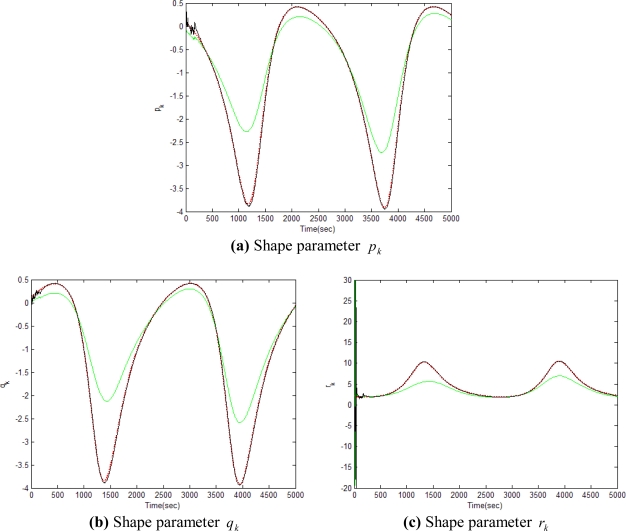
Comparison of estimation accuracy for shape parameters *p_k_*, *q_k_* and *r_k_*, for the case **b** ∈ ImΩ (true-in red; EKF- in green; UKF-in black).

**Figure 9.**
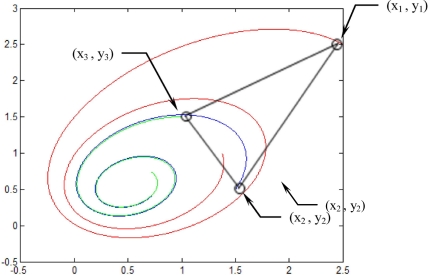
Sample trajectories for the three feature points on image plane for the case **b** ∉ ImΩ.

**Figure 10.**
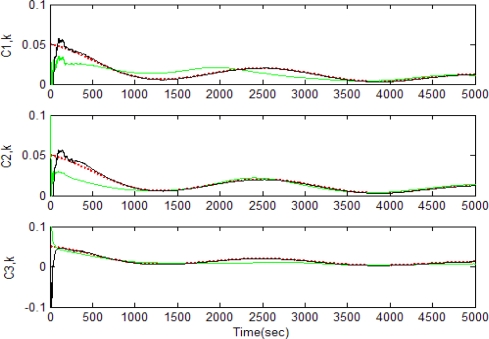
Comparison of estimation results for parameters *c*_1_, *c*_2_, and *c*_3_ for the three feature points for the case **b** ∉ ImΩ (true-in red; EKF- in green; UKF-in black).

**Figure 11.**
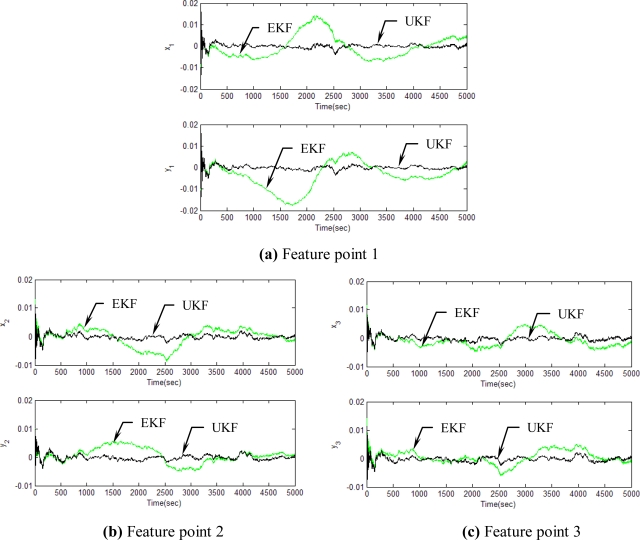
Estimation errors for the three feature points for the case **b** ∉ ImΩ.

**Figure 12.**
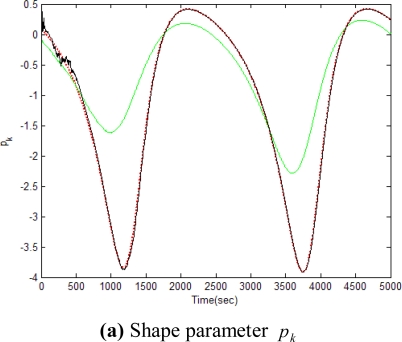

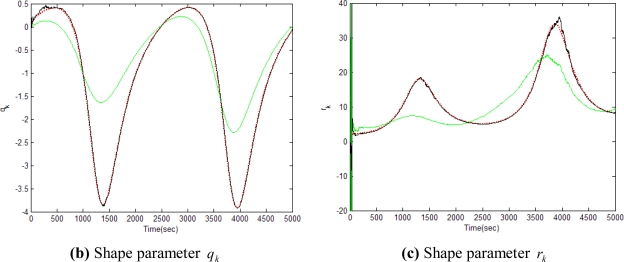
Comparison of estimation accuracy for shape parameters *p_k_*, *q_k_* and *r_k_* for the case *b* ∉ ImΩ (true-in red; EKF- in green; UKF-in black).

Figures 5–12 show the results for the numerical experiments. Figure 5 shows the sample trajectories for the three feature points on image plane for the case **b** ∈ ImΩ. It can be seen that the motions are circular and periodic around the axis of rotation. Figures 6–8 are the results for the case when **b** ∈ ImΩ. Estimation results for parameters *c*_1_, *c*_2_ and *c*_3_ for the three feature points are given in Figure 6. Three curves, shown in red, green, and black colors, denote the true, EKF-based, and UKF-based estimated values, respectively. For better clarity, the estimation errors for the three feature points are shown in Figure 7, which illustrates the advantage of UKF. Figure 8 gives the estimation accuracy for the shape parameters *p_k_*, *q_k_* and *r_k_*. The EKF is not working very well whereas the UKF demonstrates its good capability for capturing the true trajectories. Comparison of RMSE (in the unit of mm) of the three feature points on the image plane using the EKF and UKF is summarized in Table 1. Comparison of RMSE (in the unit of mm) for the feature points of the scene point using the EKF and UKF is summarized in Table 2.

Figure 9 shows the sample trajectories for the three feature points on image plane for the case **b** ∉ ImΩ. The performance comparison for EKF and UKF in the case of **b** ∉ ImΩ is basically similar to the results obtained for **b** ∈ ImΩ. The motions are receding to infinitely linearly in time. Figures 10–12 provide the results for the case when **b** ∉ ImΩ. Figure 10 shows the estimation results for parameters *c*_1_, *c*_2_ and *c*_3_and for the three feature points; Figure 11 provides the estimation errors for the three feature points; and Figure 12 gives the estimation accuracy for the shape parameters *p_k_*, *q_k_* and *r_k_*. Comparison of RMSE (in the unit of mm) of the three feature points on the image plane using the EKF and UKF is summarized in Table 3. Comparison of RMSE (in the unit of mm) for the feature points of the scene point using the EKF and UKF is summarized in Table 4.

In general, utilization of an adequate nonlinear model with suitable filter makes it possible to achieve improved estimation performance. For the problem of single camera based motion and shape estimation, the UKF-based recursive estimation algorithm is a good alternative adopted for implementing the estimation of motion and planar surface parameters. The states and the dynamic process are related nonlinearly. The nonlinearity/uncertainty in the state estimate is suitably taken care of in the UKF, which has therefore demonstrated substantial state estimation accuracy improvement as compared to the EKF based approach. When compared with EKF, the UKF method exhibits superior performance since the series approximations in the EKF algorithm can lead to poor representations of the nonlinear functions and probability distributions of interest.

## 7. Conclusions

Various engineering applications, such as robotics and machine vision, require the estimation of positions and velocities of moving features in space, leading to the problem of estimating motion and shape parameters of moving features from their corresponding image data observed over time. To alleviate the noise and obtain better estimation accuracy in feature points on the moving objects, the filtering/estimation approach can be used. A variety of estimation techniques have been adopted to reduce the noise. One of the most important ones is the extended Kalman filter (EKF).

As compared to the EKF’s linear approximation, the unscented transformation is accurate to the second order for any nonlinear function. In light of unscented Kalman filter’s superiority over the extended Kalman filter, this paper has presented a deterministic sampling of UKF approach for estimating motion and shape parameters of feature points on the moving object. Such a motion is described by the nonlinear model with skew symmetric matrix Ω, which is widely used in the theory of machine vision. The reason is due to the fact that the UKF is able to deal with the nonlinear formulation, which will ensure better accurate parameter estimation. For the nonlinear estimation problem, alternatives for the classical model-based extended Kalman filter (EKF) can be employed. The UKF is a nonlinear distribution approximation method, which uses a finite number of sigma points to propagate the probability of state distribution through the nonlinear dynamics of system. The UKF exhibits superior performance when compared with conventional EKF since the series approximations in the EKF algorithm can lead to poor representations of the nonlinear functions and probability distributions of interest.

The analyses were confirmed by simulation studies. For both cases, *b* ∈ ImΩ and *b* ∉ ImΩ, motion and shape parameters were recovered successfully with remarkable accuracy improvement. The results obtained shows that the proposed UKF method have been compared to the EKF and have demonstrated substantial improvement in obtaining the motion and shape of moving objects.

## Figures and Tables

**Table 1.** Comparison of RMSE (in the unit of mm) of the three feature points on the image plane using the EKF and UKF (**b** ∈ ImΩ).

	
	**EKF**			**UKF**		

	**x**	**y**	**x-y**	**x**	**y**	**x-y**
Feature point 1 (x1,y1)	0.0051	0.0057	0.0077	0.0011	0.0011	0.0016
Feature point 2 (x2,y2)	0.0037	0.0037	0.0052	0.0009	0.0008	0.0012
Feature point 3 (x3,y3)	0.0058	0.0043	0.0072	0.0008	0.001	0.0013

**Table 2.** Comparison of RMSE (in the unit of mm) for the feature points of the scene point using the EKF and UKF (**b** ∈ ImΩ).

	
	**EKF**				**UKF**			

	**X**	**Y**	**Z**	**X-Y-Z**	**X**	**Y**	**Z**	**X-Y-Z**
Feature point 1 (x1,y1)	5.35	5.6286	5.4815	9.5053	1.5783	1.7815	0.7082	2.4832
Feature point 2 (x2,y2)	2.4956	1.0329	1.7686	3.2284	0.621	0.2423	0.3968	0.7757
Feature point 3 (x3,y3)	1.6892	2.546	1.9205	3.6088	0.5264	0.8431	0.5515	1.1367

**Table 3.** Comparison of RMSE (in the unit of mm) of the three feature points on the image plane using the EKF and UKF (**b** ∉ ImΩ).

	
	**EKF**			**UKF**		

	**x**	**y**	**x-y**	**x**	**y**	**x-y**
Feature point 1(x1,y1)	0.0056	0.0073	0.0092	0.001	0.0011	0.0015
Feature point 2 (x2,y2)	0.0033	0.0029	0.0044	0.0009	0.0008	0.0011
Feature point 3 (x3,y3)	0.0028	0.0025	0.0037	0.0008	0.001	0.0013

**Table 4.** Comparison of RMSE (in the unit of mm) for the feature points of the scene point using the EKF and UKF (**b** ∉ ImΩ).

	
	**EKF**				**UKF**			

	**X**	**Y**	**Z**	**X-Y-Z**	**X**	**Y**	**Z**	**X-Y-Z**
Feature point 1 (x1,y1)	2.9116	2.557	5.1281	6.4276	0.476	1.4102	0.7018	1.6455
Feature point 2 (x2,y2)	0.8623	1.0275	1.6439	2.1217	0.0938	0.0894	0.1173	0.1748
Feature point 3 (x3,y3)	0.8424	1.1548	1.9177	2.3918	0.0562	0.087	0.1245	0.1619
